# Chronic alcohol administration alters metabolomic profile of murine bone marrow

**DOI:** 10.3389/fimmu.2023.1128352

**Published:** 2023-04-05

**Authors:** Tássia Tatiane Pontes Pereira, Filipe Fideles Duarte-Andrade, Jéssica Gardone Vitório, Taiane do Espírito Santo Pereira, Flavia Rayssa Braga Martins, Jéssica Amanda Marques Souza, Nathália Luisa Malacco, Eliza Mathias Melo, Carolina Raíssa Costa Picossi, Ernani Pinto, Ricardo Santiago Gomez, Mauro Martins Teixeira, Adriana Nori de Macedo, Gisele André Baptista Canuto, Frederico Marianetti Soriani

**Affiliations:** ^1^Department of Genetics, Ecology and Evolution, Universidade Federal de Minas Gerais, Belo Horizonte, Brazil; ^2^Department of Clinic, Pathology and Dental Surgery, Universidade Federal de Minas Gerais, Belo Horizonte, Brazil; ^3^Department of Analytical Chemistry of the Institute of Chemistry, Universidade Federal da Bahia, Salvador, Brazil; ^4^Department of Microbiology and Immunology, McGill University, Montreal, QC, Canada; ^5^Department of Biochemistry and Immunology, Federal University of Minas Gerais, Belo Horizonte, Brazil; ^6^Chemistry Institute, University of São Paulo, São Paulo, Brazil; ^7^Nuclear Energy Center in Agriculture, Escola Superior de Agricultura Luiz de Queiroz, University of São Paulo, Piracicaba, Brazil; ^8^Chemistry Department, Universidade Federal de Minas Gerais, Belo Horizonte, Brazil

**Keywords:** alcoholism, bone marrow, metabolome, cell function, metabolites, immune system

## Abstract

**Introduction:**

People with hazardous alcohol use are more susceptible to viral, bacterial, and fungal infections due to the effect of alcohol on immune system cell function. Metabolized ethanol reduces NAD^+^ to NADH, affecting critical metabolic pathways. Here, our aim was to investigate whether alcohol is metabolized by bone marrow cells and if it impacts the metabolic pathways of leukocyte progenitor cells. This is said to lead to a qualitative and quantitative alteration of key metabolites which may be related to the immune response.

**Methods:**

We addressed this aim by using C57BL/6 mice under chronic ethanol administration and evaluating the metabolomic profile of bone marrow total cells by gas chromatography–coupled mass spectrometry (GC–MS).

**Results:**

We identified 19 metabolites. Our data demonstrated that chronic ethanol administration alters the metabolomic profile in the bone marrow, resulting in a statistically diminished abundance of five metabolites in ethanol-treated animals: uracil, succinate, proline, nicotinamide, and tyrosine.

**Discussion:**

Our results demonstrate for the first time in the literature the effects of alcohol consumption on the metabolome content of hematopoietic tissue and open a wide range of further studies to investigate mechanisms by which alcohol compromises the cellular function of the immune system.

## 1 Introduction

Alcohol use disorder (AUD) is characterized by an impaired ability to stop or control alcohol use, despite adverse social, occupational, or health consequences. AUD is one of the most common psychiatric disorders and is a leading cause of mortality worldwide (1).

Considerable evidence indicates that alcohol abuse results in clinical abnormalities of the immune system (2, 3). Hematopoietic stem cells differentiate into myeloid progenitor cells, which are the precursor cells of granulocytes, the major type of phagocyte, constituting the front line of innate immune defense (4, 5).

Multiple lines of clinical and experimental evidence demonstrate that chronic alcohol consumption is linked to increased risks of infections, such as pneumonia. This effect has been related to alcohol’s effect on the immune system (6, 7), such as alterations in the production of bone marrow immune cells and impairment of their effector functions (3, 5).

Although there is a vast literature describing the effect of alcohol on the immune system (2, 3), there is a limitation in our understanding of the effect of alcohol on the bone marrow, and it is not known whether the cells of this system are affected by alcohol in the bloodstream or if this deleterious effect occurs inside the bone marrow microenvironment.

The majority of ingested alcohol is metabolized in the liver by hepatocytes, but immune cells such as macrophages and neutrophils can also metabolize it (8). Regardless of cell type, alcohol metabolism involves the action of alcohol dehydrogenase (ADH) and aldehyde dehydrogenase (ALDH2). The ADH enzyme is present in the cytoplasm of cells and is responsible for the oxidation reaction of ethanol that results in acetaldehyde. ADH2 is present in the mitochondria and converts acetaldehyde to acetate. These reactions involve the reduction of nicotinamide adenine dinucleotide (NAD^+^) to NADH, increasing the NADH : NAD^+^ ratio, and leading to a cellular environment vulnerable to damages caused by metabolites and adducts from ethanol metabolism and reactive oxygen species (ROS) (9).

The NADH : NAD^+^ ratio is an important parameter for the maintenance of several metabolic enzymes, and its disbalance is known to disturb cell metabolism (10), such as decreased glycolysis (11), decreased Krebs cycle (12, 13), and decreased gluconeogenesis (13, 14).

Immune cells have distinct metabolic configurations that allow them to balance energy demands and molecular biosynthesis. However, beyond that, it is now becoming clear that cellular metabolism has direct roles in regulating immune cell function, and disturbances in these metabolic configurations limit the functionality of these cells (15–17). The field of immunometabolism has advanced our understanding of how cell metabolism plays a central role in cell function, such as phagocytosis, ROS production, cell differentiation/maturation, and consequent host defenses (18–20).

In this study, we applied a metabolomic approach using gas chromatography–mass spectrometry (GC–MS) to characterize metabolic changes in the bone marrow microenvironment to test the hypothesis that alcohol could change metabolic pathways in the bone marrow and leukocyte progenitor cells, leading to a qualitative and quantitative alteration of metabolites that may be directly or indirectly related to the immune response.

We obtained a snapshot of the distinct changes in the metabolite composition of bone marrow cellular content in mice chronically exposed to ethanol. The identified metabolites suggest that chronic alcohol consumption would disrupt several metabolic pathways, such as glycolysis, the Krebs cycle, and amino acid synthesis, that could interfere with immune cell function.

Our results represent the first step toward understanding the dysfunction of the immune system due to alcohol consumption because of bone marrow microenvironment alteration of metabolite content.

## 2 Methods

### 2.1 Ethics statement and mouse model of chronic ethanol consumption

Animal experiments received approval from the Animal Ethics Committee (CEUA) of the Universidad Federal de Minas Gerais (UFMG), Brazil (Protocol 337/2018), which is in accordance with Brazilian guidelines (CONCEA) and international standards. Six-week-old male C57BL/6J mice were divided into EtOH and H_2_O groups and maintained in specific pathogen-free conditions. Animals in the EtOH group received ethanol at a rate of 5% (v/v) in the first week, followed by 10% (v/v) in the second week, and were treated for 10 weeks with 20% (v/v) of ethanol in their drinking water. The H_2_O group received water. This model, standardized by Yeligar et al. (21), generates similar blood alcohol levels to those observed in humans under chronic consumption.

### 2.2 Sample preparation for flow cytometry

Bone marrow was harvested from the femurs of six animals in each group using 0.5% BSA in 1× phosphate buffered saline (PBS). A sample for the ethanol group was lost during analysis. Total bone marrow cells were subjected to hypotonic lysis to remove residual erythrocytes. The samples were filtered in a 40 μm cell strainer, centrifuged, resuspended in 0.5% BSA in 1× PBS, fixed with 1× PBS solution containing 4% formaldehyde for 20 min, and then the cells in 0.5% BSA in 1× PBS were subjected to flow cytometry analysis on the FACSCanto II cytometer (Becton Dickinson). The relevant population was gated using accepted criteria for cell complexity and size, excluding debris and singlets (**Supplementary Figure 1**). FSC and SSC plots were assessed using FlowJo software (Tree Star, Ashland, OR, USA). Graphing and statistical analyses were performed using GraphPad Prism 8. Differences between different groups were analyzed by a student t-test.

### 2.3 Sample preparation for GC–MS

Approximately 3 × 10^7^ total bone marrow cells were obtained from three animals pooling samples for each group (H_2_O and EtOH) (n = 8 pools per group). Bone marrow was harvested from the femur and tibia using phosphate buffered saline (PBS). Red blood cells were lysed by osmotic shock. Metabolic quenching was performed using a cooling bath (dry ice/alcohol), and samples containing 1 × 10^7^ cells were centrifuged for 10 min at 225*g* at 4°C, and the completely dry cell pellet was stored in a −80°C freezer for further extraction of metabolites.

### 2.4 Metabolite extraction and GC–MS system

Metabolite extraction was performed according to modifications to the protocol described by Canuto et al. (22). Technical replicates of 1 × 10^7^ bone marrow cells were produced. Metabolites were extracted with 300 μl of extraction solvent containing methanol:chloroform:water 1:3:1 (v/v/v) followed by 2 min in a vortex mixer, four cycles of freezing and thawing in liquid nitrogen, and centrifugation for 10 min at 16,000*g* at 4°C. The entire supernatant was transferred to the glass insert and completely dried in the vacuum concentrator SpeedVac at 35°C.

Methoximation was performed by adding 20 μl of methoxyamine to pyridine (15 mg/ml). The vials were placed in an ultrasound bath for 10 s, followed by vigorous vortexing for 10 s. The samples were then incubated for 90 min at room temperature and protected from light. For silylation, 20 μl of BSTFA with 1% TCMS were added. The samples were again subjected to an ultrasound bath for 10 s, followed by vigorous vortexing for 10 s. The reaction was processed in a thermostatic bath for 30 min at 40°C. Finally, 100 μl of heptane containing an internal standard (methyl tridecanoate) was added to each sample.

Samples, QCs (quality controls), and a blank were derivatized according to the protocol described above. Samples were analyzed randomly, and QCs were analyzed at the beginning, every five samples, and at the end of the analytical sequence.

For the construction of the identification library, data were corrected for the retention times of hydrocarbon patterns (FAME MIX). Metabolites detected in the blank were removed from the final result.

The analyses were performed in a gas chromatography system (model 5975C, Agilent Technologies) coupled to a quadrupole mass spectrometer (model 7890A, Agilent Technol). A HP5-MS column (30 m, 0.25 i.d., 0.25 mm film, 95% dimethyl/5% diphenylpolysiloxane—Agilent Technologies) was used to perform the separation of the metabolites. High-purity helium was used as a mobile phase at a 1 ml/min flow rate. The injector was maintained at 250°C, and samples were injected with a 1:10 split at 10 ml/min of He. The oven was initially set at 60°C and held for 1 min, and the temperature increased to 300°C at 10°C/min, resulting in 25 min of run time.

The MS was operated in scan mode (50–600 *m/z*). An electron impact ionization source was placed at −70 eV. Detector transfer line, source filament, and quadrupole temperatures are maintained at 290, 230, and 150°C, respectively. Operation and data acquisition using Qualitative Analysis Mass Hunter B05.00 (Agilent Technologies) software.

### 2.5 GC–MS data processing and statistical analysis

Raw data were converted to *.mzData in Qualitative Analysis software (B.05.00, Agilent Technologies), and the profiles were checked for outlier removal. An optimization of data extraction and processing parameters was performed using the IPO package (isotopologue parameter optimization, version 1.16.0), using QC samples to find the best conditions. Data processing was performed in XCMS software (version 1.24.1) running on the R platform (version 3.2.3, R Core Team). The optimized parameters were as follows: “Matched Filter” method for peak detection using peak width (fwhm) = 7.2, signal/noise ratio (snthresh) = 1.0, minimum difference between m/z’s for overlapping peaks (mzdiff) = 0.36, and maximum number of peaks per extracted ion chromatogram (max) = 5. The grouping step used bandwidth correction (bw) = 0.9, width of overlapping bands of m/z (mzwid) = 0.061, minimum number of samples needed in at least one of the sample groups to be a valid group (minsamp) = 1, minimum fraction of detected samples (minfrac) = 0.5, and maximum number of peaks per extracted ion chromatogram (max) = 50 (in the first and second groupings). Alignment using retention time correction was performed using the “obiwarp” method. FillPeaks were applied to remove missing values, and the extracted molecular features (*m/z* ratios, retention times, and intensities) were normalized before statistical analysis. The raw data matrix consisted of eight samples per group, with each sample presenting the average intensity of the referred molecular feature.

Multivariate statistical analyses were performed on the MetaboAnalyst 5.0 platform, in which the data matrix of identified metabolites was normalized by the internal standard, C13 methyl tridecanoate (*m/z* 74, RT 13.73 min), and log transformation and Pareto scaling were also applied. To evaluate instrumental stability, principal component analysis (PCA) was applied, followed by partial least squares discriminant analysis (PLS-DA) to indicate metabolite differences between groups (ethanol *vs*. control), in which a VIP score >1.0 from PLS-DA was used to select discriminants.

### 2.6 Metabolite annotation

Metabolite annotation was performed in AMDIS (Automated Mass Spectral Deconvolution and Identification System) software using the Fiehn RT Library. Metabolites were annotated based on retention time and mass spectral fragmentation pattern. To do that, retention indexing followed by retention time analysis were performed. The annotated metabolites were then correlated with the raw data matrix extracted from XCMS.

## 3 Results

### 3.1 Chronic ethanol consumption does not affect the size and complexity of the bone marrow cells from mice

**Figure 1:**
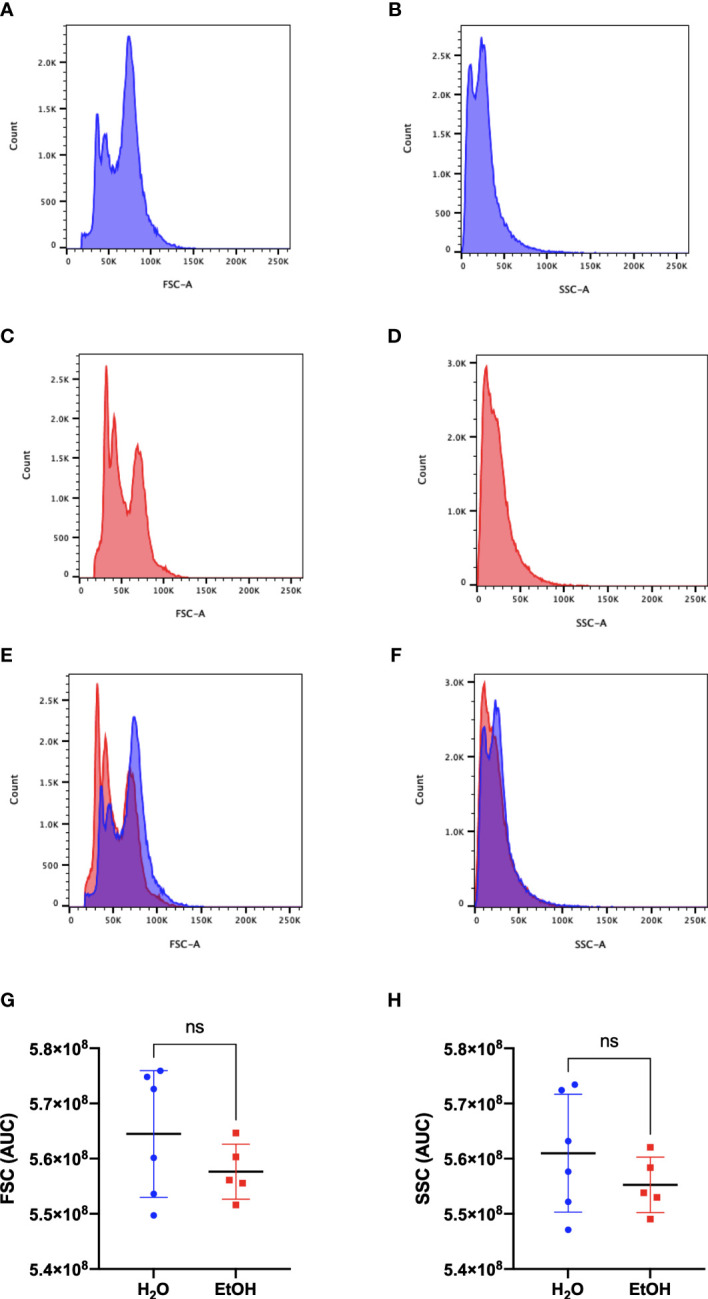
Bone Marrow cells analysis. Representative histograms of size and complexity in bone marrow from ethanol- and water-treated animals by flow cytometry. FSC-A represents the discrimination of cells by size, while SSC-A represents the complexity of cells. **(A, C)** represent the frequency of cells related to their size, and **(B, D)** represent the frequency of cells related to their complexity. **(A, B)** water-treated group **(C, D)** ethanol-treated group. **(E)** representative overlapping of **(A, C)** FSC-A graphs and **(F)** representative overlapping of **(B, D)** SSC-A graphs. In blue is the water-treated group, and in red is the ethanol-treated group. The area under the curve (AUC) of the samples was calculated and represents the distribution of the entire population used in metabolomics. A statistical analysis of AUC was conducted for FSC **(G)** and SSC **(H)**. *ns* represents not statistically significant.

To assess whether cell number is a suitable parameter for normalization of samples in the study, we evaluated the general profile of cells by flow cytometry using the complexity (SSC-A) and size (FSC-A) parameters. The gating strategy was able to select around 91% of total bone marrow cells in both experimental groups (**Supplementary Figure 1**). **Figures 1A, B** show representative FSC-A and SSC-A histograms from water-treated animals, while **Figures 1C, D** show representative FSC-A and SSC-A histograms from ethanol-treated animals. Overlapping the histograms (FSC-A in **Figure 1E** and SSC-A in **Figure 1F**) demonstrates similar distributions of cells in each parameter. To quantify the distribution of sizes and complexities of cells in both groups, we analyzed the area under the curve (AUC) and the results demonstrated that ethanol treatment did not change the distribution of cell sizes (**Figure 1G**) or cell complexities (**Figure 1H**) in the bone marrow.

### 3.2 Chronic alcohol consumption alters the metabolomics profile in the bone marrow

**Figure 2:**
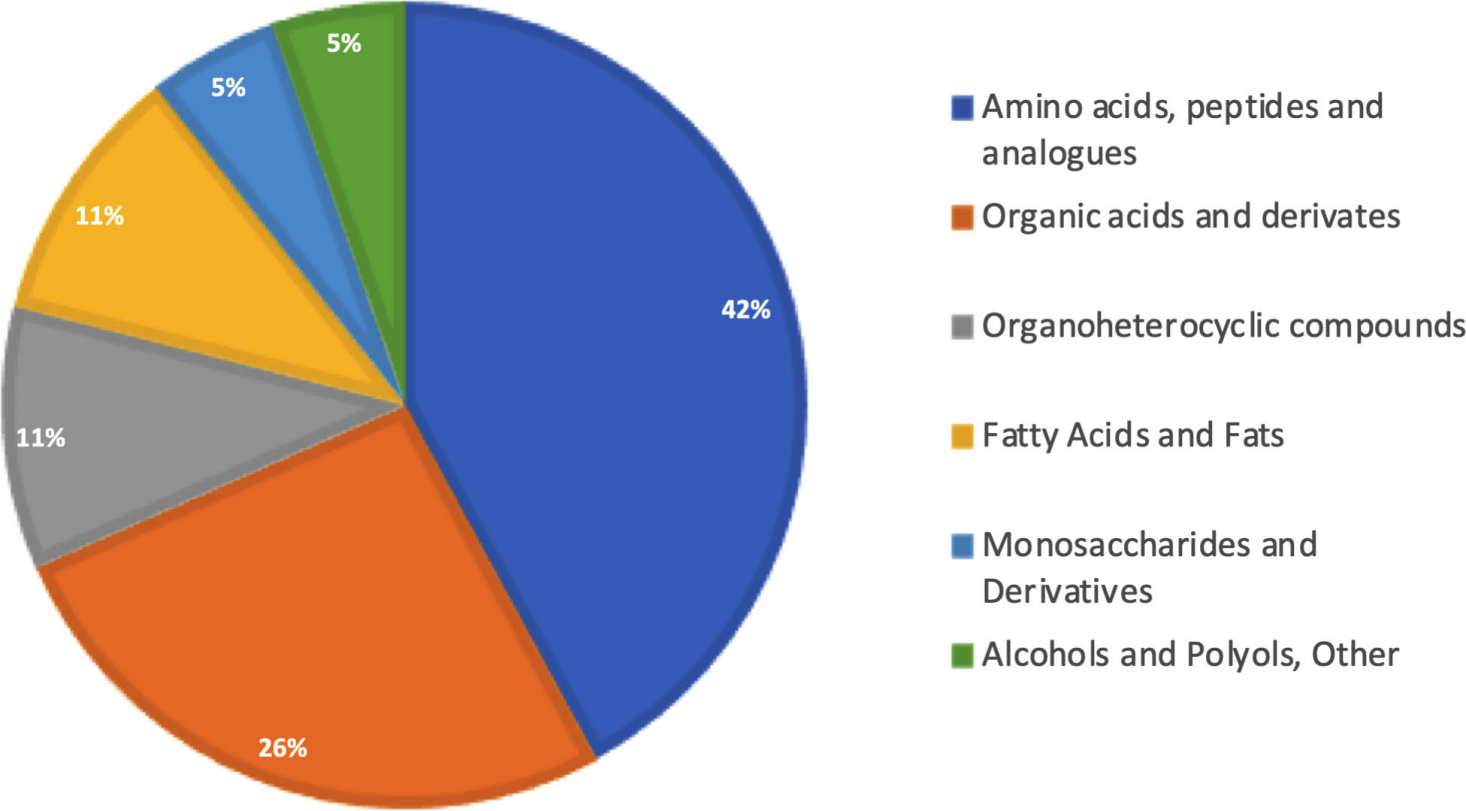
Biological categorization of identified metabolites. All 19 identified metabolites were classified according to functional categorization in PubChem (https://pubchem.ncbi.nlm.nih.gov). Percentages represent the number of classified metabolites in each category.

Gas chromatography–coupled mass spectrometry was performed using pooled bone marrow cells. After carrying out the identification processes in AMDIS, correlation with the matrix extracted from XCMS, and removal of the analytes present in the blank, our approach was effective in identifying 19 metabolites (**Table 1**) that were classified into six different Gene Ontology classes: amino acids, organohetetocyclic compounds, monosaccharides, organic acids, and fatty acids alcohols/polyols (**Figure 2**). Metabolites 3 and 17 present two possibilities for identification.

**Table 1:** Metabolites identified by GC–MS analysis and statistically significant altered metabolites in bone marrow cell of ethanol-treated mice.

Metabolite	VIP score	FC (EtOH/H_2_O)	Chemical Classification
1. Valine	0.63	0.94	Amino acids, peptides, and analogues
2. Alanine	0.99	0.97	Amino acids, peptides, and analogues
3. Leucine/isoleucine	0.06	0.95	Amino acids, peptides, and analogues
4. Benzoic acid	0.62	0.51	Organic acids and derivates
5. Serine	0.82	0.87	Amino acids, peptides, and analogues
**6. Proline**	**1.26**	**0.98**	**Amino acids, peptides, and analogues**
**7. Succinic acid**	**1.38**	**0.89**	**Organic acids and derivates**
**8. Uracil**	**2.90**	**0.71**	**Organoheterocyclic compounds**
9. Fumaric acid	0.54	0.91	Organic acids and derivates
10. Aspartic acid	0.73	0.97	Amino acids, peptides, and analogues
**11. Nicotinamide**	**1.20**	**0.70**	**Organoheterocyclic compounds**
12. Malic acid	0.28	0.96	Organic acids and derivates
13. Glutamic acid	0.91	0.91	Amino acids, peptides, and analogues
14. Lauric acid	0.28	0.87	Organic acids and derivates
**15. Tyrosine**	**1.07**	**0.86**	**Amino acids, peptides, and analogues**
16. Hexadecanol	0.01	0.77	Alcohols and Polyols, Other
17. Mannitol/altrose	0.64	1.16	Monosaccharides and Derivatives
18. Linoleic acid	0.34	0.72	Fatty Acids and Fats
19. Oleic acid	0.36	1,11	Fatty Acids and Fats

FC, Fold Change; VIP score, Varial Importance in Projection.

The metabolites present in lines 3 and 17 present two possible identifications. This is due to the analytical impossibility of differentiating these isomers as a function of elution at very close retention times and because they present identical MS fragmentation profiles. Therefore, the identification is presented with both isomers. Statistically significant altered metabolites that are present are shown in bold.

**Figure 3:**
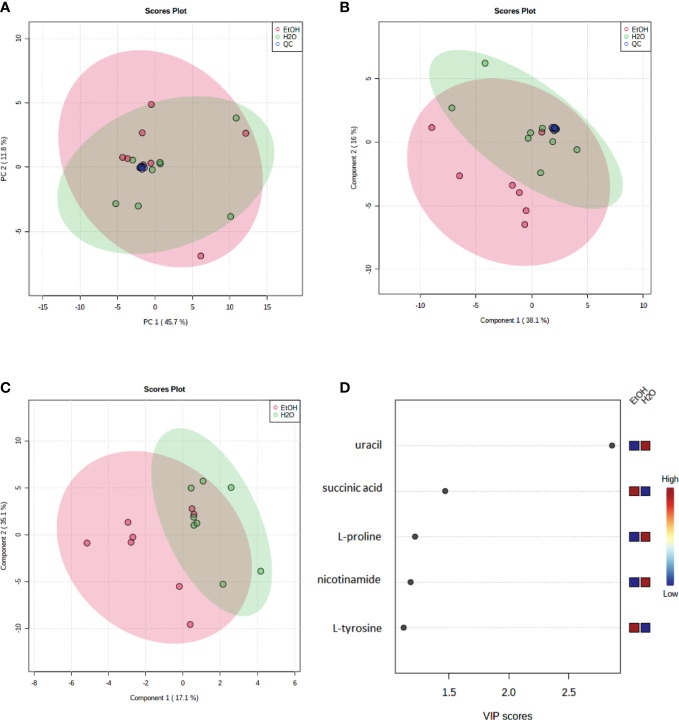
Multivariate models (Pareto scaling) of metabolites identified in cell samples analyzed by GC–MS and discriminating metabolites between ethanol-treated and control groups by GC–MS. **(A)** Principal component analysis (PCA) and **(B)** partial least squares discriminant analysis (PLS-DA). Each dot represents one sample, and **(A, B)** show a separation of the groups and a robust clustering of the Quality Controls (QC). PSL-DA model parameters: R2 = 0.58 and Q2 = 0.26). Quality controls (QC—blue dots), a water-treated control group (H_2_O—green dots), and ethanol-treated experimental group (EtOH—red dots). **(C)** The PLS-DA Model (Pareto scale) shows a separation between the groups; **(D)** the VIP Score Chart contains the discriminating metabolites between groups. A VIP score >1 is considered significant. A red (high) to blue (low) scale indicates the relative abundance of metabolites in the ethanol-treated group compared to the water-treated control group. The green ellipses represent the water-treated control group, and the red ellipses represent the ethanol-treated experimental group.

To evaluate the instrumental performance, principal component analysis (PCA) (**Figure 3A**) and partial least squares discriminant analysis (PLS-DA) (**Figure 3B**) was applied, including the QC samples. The supervised model (PLS-DA) was validated using a distance separation method with 100 permutations, considering a p-value of ≤0.05. It is possible to observe an excellent group of QCs demonstrating the quality and reliability of the instrument for data acquisition.

Moreover, to better identify and discriminate metabolites, a new model (PLS-DA) (**Figure 3C**) was conducted without the QC samples, and a VIP score >1.0 (**Figure 3D**) was used to consider statistically significant differences in abundance of the metabolites between groups. Five metabolites were identified with statistical significance by multivariate analysis (uracil, L-tyrosine, L-proline, succinic acid, and nicotinamide).

**Table 1** presents, in bold, the significantly altered metabolites between groups comparison, in which the statistical results (VIP score) and the variation rate fold change (FC) are presented. These five statistically different abundance metabolites are decreased in the ethanol-treated group at several intensities.

## 4 Discussion

Metabolic profiles have been explored in many diseases (23), including liver diseases resulting from alcoholism (24–27). This study was the first to investigate the impact of chronic alcohol consumption on the bone marrow metabolic profile using an *in vivo* model of chronic ethanol consumption. The motivation for this study stems from evidence demonstrating that chronic consumption has negative effects on the ability of individuals with hazardous alcohol use to respond properly during infections (2, 28, 29).

Analytical tools for metabolomics studies have high sensitivity, being able to identify and quantify the presence of analytes at low concentrations (30). Therefore, the first step of the present study was to evaluate, by flow cytometry, whether the parameter number of cells would be adequate, since alcohol could be causing an increase in cell size, resulting in a bias toward greater abundance of the metabolite. The descriptive evaluation obtained from the FSC, and SSC histograms showed that ethanol treatment does not cause changes in the size/volume of cells in the bone marrow, ensuring that the quantitative differences found in this study are because of alcohol on metabolic pathways, resulting in a distinct metabolic profile.

The negative effect of chronic ethanol consumption on the immune system and bone marrow has already been investigated. It is well established that the cells of the immune system of individuals affected by alcoholism have a lower capacity to migrate to the infectious site, phagocyte, and eliminate the pathogen, and the mechanism is often related to the production of cytokines and chemokines that have their levels and activities affected (21, 31–34). In the bone marrow, studies have shown that alcohol consumption promotes important effects on hematopoiesis (35, 36).

In this study, we evaluated the effect of ethanol on bone marrow at the level of metabolites. Once cellular metabolism plays an important role in the functionality of immune cells, changes in the metabolic profile can compromise their functionality (15).

The metabolomics profile revealed significant effects of chronic alcohol consumption on the metabolome of mouse bone marrow. Although the analytical restrictions of the gas chromatography approach limit the scope of the study to volatile compounds and/or volatile compounds through derivatization, it was possible to obtain a holistic approach to the metabolic profile of this tissue as well as the changes resulting from chronic exposure to alcohol.

Nicotinamide (C_6_H_6_N_2_O) showed reduced abundance in cells from animals under chronic treatment with ethanol. Nicotinamide is the active form of vitamin B3 and a component of the coenzyme nicotinamide adenine dinucleotide (NAD). When ethanol is metabolized, it generates a reduced cellular environment due to the use of nicotinamide adenine dinucleotide (NAD^+^) as an enzymatic cofactor at both stages of its metabolism. The reduced cellular environment has been related to the dysfunctions observed in the cells of the immune system (2, 21, 37, 38). In addition, NAD^+^ is an enzyme cofactor used in important metabolic pathways such as glycolysis and the Krebs cycle, which have been reported to be essential for neutrophil and macrophage function (15, 39, 40). We hypothesize that ethanol metabolism limits the availability of NAD^+^ for cell metabolism, altering the metabolic profile. This alteration may be related to the negative effect of alcohol on the function of these cells. Our results suggest that this may be part of the mechanism by which alcohol alters metabolism in this tissue. Although functional studies are needed to confirm and elucidate this evidence.

Succinic acid, an important component of the TCA cycle, was recently identified as a modulator of the innate immune response. In lipopolysaccharide (LPS)-activated macrophages, succinate was identified as a key metabolite in innate immune response signaling since its increase is correlated to increased production of interleukin-1b during inflammation (41, 42). Furthermore, lipopolysaccharide-induced succinate stabilizes hypoxia-inducible factor (HIF-1α), an effect that is inhibited by 2-deoxyglucose, with interleukin-1b as an important target (41, 43). HIF-1α is an oxygen-dependent transcriptional activator that plays crucial roles in tumor angiogenesis and mammalian development (44). Furthermore, HIF-1α increases macrophage aggregation, invasion, and motility and boosts the expression of pro-inflammatory cytokines. HIF-1α also increases neutrophil survival by inhibiting apoptosis and triggering NF-κB-dependent neutrophilic inflammation (45). Succinate was also shown to promote hematopoietic cell proliferation by phosphorylation of the ERK1/2 mitogen-activated protein kinase (MAPK) pathway and inositol phosphate accumulation in a pertussis toxin (PTX)-sensitive manner (46). Furthermore, succinate induced activation of ERK1/2, JNK, and p38 MAPK signaling pathways in immortalized retinal ganglion cells (RGC-5) cells in a dose-dependent manner (47). The ERK1/2 and MAPK pathways are related to the activation of the pro-inflammatory response of immune cells (48–50). Therefore, the deregulation in the amount of succinate found in this work can be considered a modulating mechanism of chronic alcohol consumption in the immune response.

In agreement with studies that investigated changes in the metabolome associated with alcohol consumption in humans, amino acids are the most representative chemical class (51). Tyrosine and proline are non-essential amino acids used in protein biosynthesis. Protein tyrosine (PTP) phosphorylation is an important post-translational modification that controls cell signaling involved in the regulation of a variety of biological processes, including cell growth, proliferation, differentiation, migration, survival, and death. The negative effect of downregulating these amino acids in the ethanol-treated group can impair an important biological process since tyrosine phosphorylation is considered one of the fundamental steps in signal transduction and regulation of enzymatic activity (52). However, studies that investigate the relationship between quantitative alterations of amino acids and the function of immune system cells were not found in the literature. The results found here suggest that investigations in this direction may be promising for understanding the mechanism behind the harmful effect of alcohol on immune response. Once some amino acids like tyrosine can be catabolized all the way down into intermediaries of the Krebs cycle, especially into fumarate and acetoacetate (53).

Uracil is a common natural pyrimidine found in RNA and was found to be downregulated in the bone marrow of ethanol-treated mice. Despite not finding in the literature a direct relationship between this nucleotide and the function of immune cells, it is known that uracil helps to carry out the synthesis of many enzymes necessary for cell function through the interaction with ribose and phosphates and serves as an allosteric regulator and a coenzyme for many important biochemical reactions (54).

A wide variety of types of RNAs act in the regulation of the immune system. microRNAs, RNA-binding proteins that control the stability and translation of messenger RNA (mRNA) and RNA interference (RNAi) are examples of RNAs that act by controlling the gene expression of cytokines and chemokines responsible for the intercellular communication of the immune system (55, 56). The decrease in uracil levels resulting from the chronic consumption of ethanol in the bone marrow may indirectly compromise the entire elaboration of the immune response.

Metabolic profiles can be considered a phenotypic state that undergoes variations under the influence of changes in the genome, proteome, transcriptome, metabolism, and modifications in the microenvironment where they are found (57). In our approach, we demonstrate for the first time that chronic ethanol consumption can alter the bone marrow microenvironment, and this can be associated with altered immune cell metabolism, leading to a programmed alteration function of the mature circulating cells. However, this is an initial, exploratory metabolomics study that brings important insights into cellular metabolism under alcohol exposure. We are aware of some limitations of our approach, such as the influence of animal gender in metabolism and susceptibility to alcohol, the use of other analytical platforms that should improve the number of identified metabolites, and the need for functional studies to demonstrate the role of metabolic pathways in the immune response. In addition, the effects of blood alcohol concentration and the effects of the products of its metabolism (e.g., acetaldehyde) can also be considered. Complementary studies will allow greater coverage of the metabolome, in addition to confirming and validating the hypotheses raised in this work.

## Data availability statement

The original contributions presented in the study are included in the article/**Supplementary Material**. Further inquiries can be directed to the corresponding author.

## Ethics statement

The animal study was reviewed and approved by Animal Ethics Committee (CEUA) of Universidad Federal de Minas Gerais (UFMG), Brazil (Protocol 337/2018) which are in accordance to the Brazilian guidelines (CONCEA) and international standards.

## Author contributions

Conception of the study: FS. Designed the experiments: TP, FA, JV, and FS. Performed the experiments: TP, JS, FM, CP, and EP. Interpretation of the results and data analysis: TP, TS, GC, AM, NM, and FS. Contributed reagents/materials/analysis tools: MT, FS, and RG. Wrote the manuscript: TP and FS. Helped in animal experiments: EM. All authors contributed to the article and approved the submitted version.
