# Supplementary figures and images for: Chronic alcohol administration alters metabolomic profile of murine bone marrow
Source: Front Immunol. 2023 Apr 5;14:1128352. doi: 10.3389/fimmu.2023.1128352 (PMC10113543; doi:10.3389/fimmu.2023.1128352)

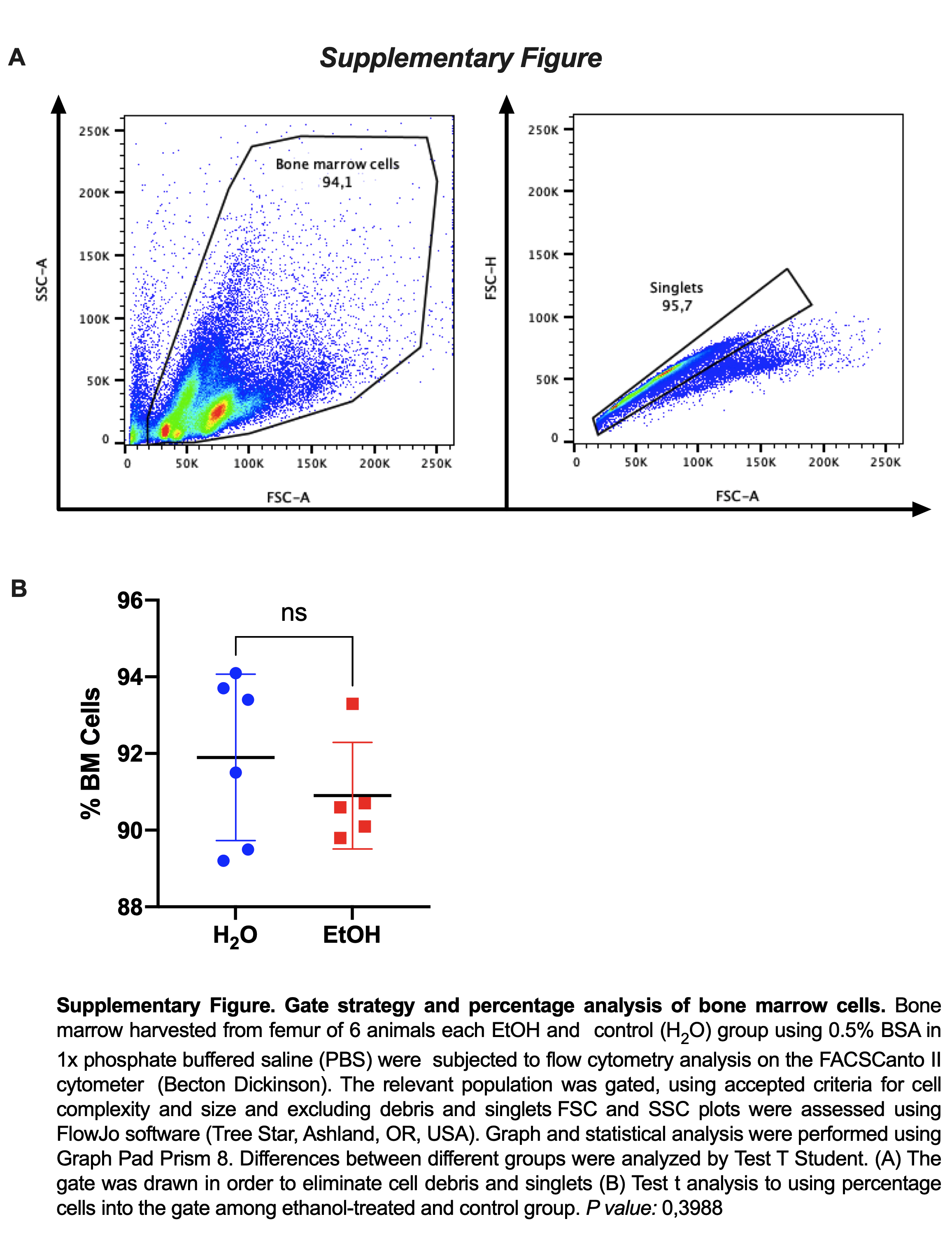

Supplement: Supplementary file 1 [file Image_1.tiff]
